# Integrating PrEP delivery in public health family planning clinics: a protocol for a pragmatic stepped wedge cluster randomized trial in Kenya

**DOI:** 10.1186/s43058-021-00228-4

**Published:** 2021-12-11

**Authors:** Kenneth K. Mugwanya, Daniel Matemo, Caitlin W. Scoville, Kristin M. Beima-Sofie, Allison Meisner, Dickens Onyango, Mary Mugambi, Erika Feutz, Cole Grabow, Ruanne Barnabas, Bryan Weiner, Jared M. Baeten, John Kinuthia, for the FP Plus Team

**Affiliations:** 1 Departments of Global Health and Epidemiology, University of Washington, 325 Ninth Avenue, #HMC 359927, Seattle, WA 98104 USA; 2 Research & Programs, Kenyatta National Hospital, Nairobi, Kenya; 3 Department of Global Health, University of Washington, Seattle, USA; 4 Vaccine and Infectious Diseases Division, Fred Hutchinson Cancer Research Center, Seattle, USA; 5 Kisumu County Department of Health, Kisumu, Kenya; 6 National AIDS and STI Control Program Ministry of Health, Nairobi, Kenya; 7 Departments of Global Health and Medicine, University of Washington, Seattle, USA; 8 Departments of Global Health, Medicine, and Epidemiology, University of Washington, Seattle, USA; 9 Present affiliation: Gilead Sciences, Foster City, USA; 10 Kenyatta National Referral Hospital, Nairobi, Kenya

## Abstract

**Background:**

Adolescent girls and young women account for a disproportionate fraction of new HIV infections in Africa and are a priority population for HIV prevention, including provision of pre-exposure prophylaxis (PrEP). Anchoring PrEP delivery to care settings like family planning (FP) services that women already access routinely may offer an efficient platform to reach HIV at-risk women. However, context-specific implementation science evaluation is needed.

**Methods:**

The Family Planning Plus Project is a prospective, pragmatic implementation evaluation, designed as a stepped wedge, cluster randomized trial, at 12 clinics in Kenya. In collaboration with the Kenya Ministry of Health and Kisumu County Department of Health, we will introduce integration of HIV risk screening and PrEP delivery in public health FP clinics. The core multifaceted implementation strategies to integrate PrEP in FP clinics will include: (1) PrEP delivery by existing FP clinic staff, (2) health provider training, (3) PrEP technical assistance to coach and mentor providers, (4) joint supervision with Kisumu County health officials, and (5) stakeholder engagement. All core components of PrEP delivery—including screening for HIV risk, HIV testing, dispensing, adherence and risk reduction counseling, assessment of side effects, and provision of refills, or safety assessment—will be conducted by existing FP clinic staff as part of a standard care service package. The goal is to catalyze sustainable scale-up within existing infrastructures beyond the project. We will rigorously evaluate implementation outcomes and impact, using the RE-AIM (Reach, Effectiveness, Adoption, Implementation, Maintenance) framework, and we will use Organizational Readiness for Implementing Change (ORIC) and the Consolidated Framework for Implementation Science Research (CFIR) to assess readiness to implement and contextual enablers and barriers of implementation, including how clinics innovate efficient delivery systems.

**Discussion:**

Anchoring PrEP delivery to existing FP systems and staffing has tremendous potential to address barriers that women face in accessing HIV prevention and PrEP care, including lack of time, cost, and stigma of visiting a facility solely for HIV prevention. The FP Plus Project will initiate preparation for full-scale and sustainable model of integration of comprehensive HIV prevention services, including PrEP implementation, in public health FP clinics in low-income settings.

Trial registration

Registered with ClinicalTrials.gov on December 14, 2020: NCT04666792

Contributions to the literature

- Young African women are a priority for HIV prevention including PrEP provision because they account for a disproportionate percentage of new HIV infections.
- Routine family planning clinics are attractive settings for PrEP implementation due to broad coverage for sexually active women. However, there is limited real-world evidence on effective strategies for integrated family planning and PrEP delivery in African settings.
- Rigorous implementation science evaluations from this work will provide important information on how to effectively and sustainably reach at-risk women for PrEP in resource-limited settings. The findings will also be valuable in translating emerging HIV prevention modalities into real-world implementation.

## Background

Women account for a disproportionate percentage of individuals with new HIV infections in Africa and are a priority population for HIV prevention [1], including provision of pre-exposure prophylaxis (PrEP). PrEP is a highly effective user-controlled prevention method, with potential to reduce incident HIV infections in Africa if delivered with sufficient coverage among at-risk populations [2–4]. In many settings in Africa, family planning (FP) clinics provide broad coverage for women in their reproductive years [5] and offer an opportunity to integrate HIV prevention and sexual and reproductive health services (SRH), including PrEP provision and management of sexually transmitted infections (STIs). A recent large clinical trial of contraceptive use and HIV acquisition (ECHO Trial) found that HIV risk was alarmingly high for women seeking family planning [6]. These results have emphasized the need to strengthen integration of HIV prevention and FP services. The World Health Organization (WHO) has issued a renewed call-to-action for efforts and commitment to support countries with high HIV incidence rates, to develop plans to provide integrated FP and HIV and STI services [7].

In the recently completed PrEP Implementation in Young Women and Adolescent (PrIYA) program (funded through PEPFAR DREAMS innovation challenge), we pioneered PrEP provision in FP clinics and demonstrated feasibility of integrating PrEP provision in FP systems using project-dedicated staff embedded in FP clinics [8]. Over 1271 women seeking FP services were screened for HIV risk factors across eight FP clinics in Kisumu County, Kenya with 22% PrEP uptake in during a 12-month program period. However, PrEP uptake declined when project-dedicated staff left after the end of the program, demonstrating the need for sustainable PrEP delivery models that fully integrate into existing staff and clinic systems. With more efficient PrEP integration, there is opportunity to optimize women-centered care with a one-stop approach to facility-based FP and HIV prevention care. Here, we describe the design and the application of the Reach, Effectiveness, Adoption, Implementation, and Maintenance (RE-AIM), Organizational Readiness for Implementing Change (ORIC), and the Consolidated Framework for Implementation Science Research (CFIR) frameworks to a programmatic stepped-wedge evaluation to integrate PrEP in Kenyan public health FP systems (the FP Plus Project). Triangulation of data from multiple sources guided by these frameworks will help to characterize key processes needed for the development of sustainable integration of FP and HIV prevention services.

## Methods

### Overall goal and specific aims

The overall goal of the FP Plus Project is to catalyze integration of comprehensive screening for HIV risk and PrEP delivery in public health FP clinics in Kenya. The specific aims are:

1) Facilitate integration of comprehensive screening for HIV risk behaviors and delivery of PrEP in public FP clinics and evaluate program impact.

2) Assess clinic readiness to implement PrEP, fidelity to PrEP service delivery, impact on current services, and facilitators and barriers to integrating PrEP delivery in FP clinics

3) Assess incremental costs, budget impact, and affordability of integrating PrEP delivery in FP clinics

4) Refine and optimize simple data systems that will expand and support delivery of integrated FP and HIV prevention services at scale.

### Population, Setting, and Facility selection

The population includes sexually active HIV-uninfected women of reproductive years accessing FP services in the Kisumu area of Kenya, given it is a high HIV burden area [9]. The project is conducted at 12 facilities selected through a joint decision process by project investigators and Kisumu County officials. Clinic selection was based on patient volume, readiness to participate, and catchment area to capture the diversity that represent the geographic, economic, and demographic characteristics of all women accessing FP services and breadth among the clinic staff.

Eligibility to participate in the program will reflect the real-world implementation nature of this work. Thus, the program will be set up for all women of reproductive age ( ≥15 years), HIV uninfected or of unknown HIV status receiving routine FP services. The broad eligibility criteria is designed to align with the public health nature of this work; to operate effectively, efficiently, and ethically in community settings, PrEP will need to be broadly accessible to all at-risk adolescent girls and young women who shoulder the biggest burden of HIV infections in this setting. Determination of eligibility for PrEP will be conducted according to the Kenya national PrEP guidelines. All women determined to be at risk for HIV, or expressing interest in PrEP, will have access to PrEP medication free of charge as part of the Kenya national PrEP scale-up program.

### Project Design and Intervention

**Fig. 1:**
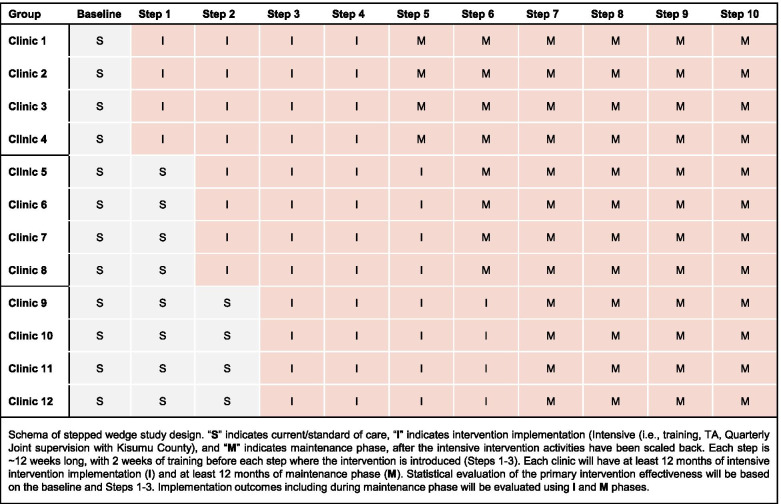
Schematic of stepped-wedge cluster randomized trial design.

The FP Plus Project is a prospective, pragmatic stepped wedge cluster randomized trial (SWCRT). In collaboration with the Kenya MOH and Kisumu County department of health, systematic screening for HIV risk and counseling for and provision of PrEP will be introduced and actively promoted in FP clinics in three staged successive waves (steps), occurring approximately every three months with an average of 60 HIV-uninfected women accessing services per clinic per step, and four clinics initiating implementation per step (Fig. 1). We will use steps four to ten (Fig. 1) to refine and optimize the intervention with varying ongoing technical assistance and supervision intensity to understand and document implementation process and outcomes and how effectively delivery of integrated PrEP services is institutionalized in FP clinics.

### Randomization

Randomization was carried out at the beginning i.e., a single point in time (at a public virtual meeting with the Kisumu County Health leadership and health facility representatives), randomizing the order with which the clinics (clusters) cross over from usual care to implementation of the intervention package. Randomization was conducted without restriction and implemented in two-stages using an online wheel-of- fortune. First, clinics were assigned to three groups of four clinics each and second, the groups were then assigned to the order (i.e., order of steps) to which they would start implementation (Fig. 1).

### Intervention: Implementation strategies to integrate PrEP services in FP clinics

For this implementation project, the intervention to integrate PrEP in Kenyan FP clinics is a multifaceted implementation strategy with the following core components: 1) promotion of comprehensive screening for HIV risk and PrEP eligibility by existing FP staff (including HIV testing, HIV risk screening, male partner testing, PrEP provision, and adherence and safety counseling); 2) Training of existing health care providers; 3) PrEP technical assistance to coach and mentor health providers; and 4) Joint supervision with Kisumu County health officials. At all implementing clinics, we will conduct a pre-implementation needs assessment to understand and document current processes on HIV risk screening and testing, existing PrEP screening and provision (expected to be minimal), STI screening, testing and treatment, and male partner testing. Prior to initiating implementation at each clinic, we will conduct clinic-wide training for health care providers, and we will engage existing FP providers in ongoing continuous quality improvement to optimize PrEP delivery. Clinics will start implementation after two weeks of intensive training. Each clinic will have at least 12 months of intensive implementation of the intervention package followed by at least 12 months of maintenance phase (Fig. 1)

#### Screening for HIV risk and PrEP provision

We will optimize integration of comprehensive HIV prevention services and PrEP delivery using existing FP clinic providers within existing infrastructure. All core components of PrEP delivery– including screening for HIV behavior risk, HIV testing, dispensing, adherence and risk reduction counseling, assessment of side effects, provision of PrEP refills, and safety assessments – will be conducted by existing FP clinic staff as part of the standard of care service package. The goal is to catalyze scale-up within existing clinic infrastructure that is sustainable beyond the study. As part of standard of care services, clinics will promote comprehensive provision of integrated FP services and HIV prevention services, including contraception methods use and preferences, promotion of knowledge of partner HIV status, condom provision, and screening and treatment of STIs. Determination of eligibility for PrEP, clinical provision of PrEP, and follow-up of clients who initiate PrEP will be conducted according to the Kenya national PrEP guidelines using MOH/NASCOP tools, which include a risk assessment screening tool and clinical encounter form [10]. The Kenya PrEP guidelines identify the presence of any of the following behavioral factors in the last six months as indication for substantial ongoing risk of acquiring HIV: 1) Inconsistent or no condom use, 2) Having high risk sex partner(s) and of unknown HIV status, 3) Engaging in transactional sex, 4) History of ongoing intimate partner and gender-based violence, 5) Recent sexually transmitted infections- self-reported or etiologically diagnosed, 6) Recurrent use of post-exposure prophylaxis, 7) Recurrent sex under the influence of alcohol/recreational drugs; 8) Injection drug use with shared needles and/or syringes, and 9) Having an HIV positive partner. Per the Kenya MOH PrEP guidelines, persons who initiate PrEP have follow up visits at one month and then quarterly after initiation, with HIV testing, ongoing behavior risk assessment, and adherence and safety counseling.

PrEP medications and commodities will be provided by the Kenya national stock of medications from the Kenya Medical Supply Authority as part of the national PrEP scale-up program. PrEP has regulatory and normative guidance sanctions in Kenya and is defined as part of standard care, in part based on a series of clinical trials and implementation studies done in Kenyan populations [11–20]. All women determined to be at risk for HIV, or expressing interest in PrEP, will receive PrEP medication from FP clinics free of charge as part of the Kenya national PrEP scale-up program.

#### Provider training to strengthen capacity for PrEP implementation

The program will work with the MOH/NASCOP and Kisumu County Health Team to support clinics’ readiness to deliver PrEP in a combination HIV prevention package in FP clinics. Provider training will be conducted at each of the participating clinics (in-facility training) using the approved case-based interactive Kenya MOH/NASCOP PrEP training curriculum [21]. Content specific to FP clinics will be added, including topics on FP, STI, treatment, and partner testing services with the goal of equipping providers with the comprehensive knowledge and skills to provide integrated FP and combination HIV prevention services, including PrEP services. The training will be conducted at each facility and curriculum modules will be delivered over a 2-week period immediately prior to implementation. A Continuous Medical Education-like refresher training will be delivered quarterly following implementation. Clinics will begin PrEP delivery within two weeks of training.

#### PrEP technical assistance

We will conduct ongoing technical assistance to clinics using trained experienced program nurses to coach, mentor, and guide FP clinics to troubleshoot emerging challenges with integrating new services. Technical advisors (TAs) will be project nurses with training in PrEP delivery. TAs will conduct structured periodic visits to each clinic to engage with the health care providers and observe and document PrEP implementation processes, with a specific focus on delivery modifications over time to understand integration optimization within and between facilities. Using rapid-cycle analysis approaches, TAs will make summary reports at the end of each clinic visit highlighting what changed, why it changed, who initiated the changes, and outcome of any changes instituted. Best practices will be shared across all clinics (cross-clinic pollination) for possible adoption. The project will work with the County Health team and implementing clinics to optimize simple data systems that include indicators for screening HIV risk and PrEP provision.

#### Joint supervision with Kisumu County health team

At each clinic, the TAs will visit the clinic weekly for first three months, and then complete monthly visits through the 12-month intensive implementation phase. During the maintenance phase, TAs will complete quarterly visits or as requested by the implementing clinics focused on understating institutionalization of integration. Every three months throughout the first 18 months of intervention implementation (i.e., 12 months of intense implementation and at least 6 months of maintenance), the TAs will be accompanied to the facility visits by County health and or Kenya MOH/NASCOP supervisors to audit for process and integration indictors for FP services, HIV prevention services, and PrEP and progress.

### Sample size and power

The co-primary outcomes will be the proportion of women screened for HIV risk and PrEP eligibility and the number and proportion of women eligible women who initiate PrEP. The study has been powered for the PrEP eligibility screening outcome. The denominator will consist of all HIV uninfected women accessing services at the implementing FP clinic during each step (time period). Although screening for HIV risk behaviors is an expected service in FP clinics, it is not routinely done or documented in most FP settings. After the intervention, we expect to see screening for HIV risk and PrEP among general population women to increase from ~5% to 25% (mean ~20%). We used an intracluster correlation coefficient value of 0.15, which is generally considered conservative in this context, and we conservatively allowed the proportion of HIV-uninfected women counseled on HIV risk and PrEP to be 6% in the absence of intervention at the end of the study (i.e., a 20% increase in proportion counseled due to factors outside of the intervention). Given these parameters, if we enroll 56 HIV-uninfected women in each of the 12 clinics during each of the first four steps (including the baseline step), where four clinics initiate the intervention in each of first three post-baseline steps (yielding 2688 total participants across the 12 clinics and the first four steps), we would have 80% power. Note that these calculations provide the number of HIV-uninfected women required; the number of women needing to be screened would be higher (and would depend on the HIV positivity rate). To fully understand how clinics build new efficient systems for delivery of integrated FP and HIV prevention services, we will study data on a total of at least 5000 women accessing routine services across the 12 FP clinics. This sample size will permit a full-scale test of the system, since implementation outcomes and the efficiency in delivery are to a greater part at the system level, above and beyond the individual client encounter.

### Data collection, measurement, and evaluation of process and implementation outcomes

We will use multiple data sources (Table 1) to robustly synthesize and triangulate the process of integrating PrEP delivery in public health FP clinics and to identify contextually relevant strategies for successful implementation. Data sources will include: 1) Abstraction of program data, 2) Qualitative interviews (policy key informants, healthcare providers, women), 3) Quantitative questionnaires (healthcare providers, women), 4) TA visit summaries and rapid cycle debrief reports, 5) Patient actors, 6) Clinic and client flow mapping, 7) Activity-based costing and time and motion studies; 8) individual-level clinical data from the nested observational cohort, and 9) Random blood draw for adherence (Table 1).

**Table 1:** Data sources

Data source	Description	Purpose
Data abstraction	▪ Data abstracted from clinical delivery tools	▪ Define profile of screened and initiated on PrEP and whether persons are appropriately put on PrEP
Technical assistance reports and rapid cycle debriefs.	▪ Rapid cycle debriefs and TA reports prepared at baseline and 6-monthly	▪ Document detailed knowledge of process of integration of PrEP delivery and track changes in PrEP implementation processes ▪ Rapid cycle analysis to convey to facilities for quality improvement
Qualitative interviews: women, healthcare providers, and policy key informants	▪ Purposefully sampled client and key informants involved in the delivery	▪ Characterize process of adoption and integration of PrEP delivery and track changes in PrEP implementation processes, including barriers and facilities
Quantitative surveys	▪ Healthcare providers ▪ Random cross-sectional exit surveys with women at the end of clinic visit	▪ Assess acceptability, clinics’ readiness to implement, clinic inner settings ▪ HIV and STI risk perception, characteristics of women accessing through FP clinics, stigma, and satisfaction with services offered.
Activity-based costing, and time and motions studies	▪ Primary data collection	▪ Activity-based costing, and time and motions studies
Clinic and client flow mapping	▪ Primary data collection	▪ Establish baseline flow, track how new services are incorporated in the flow and bottlenecks, and document patient wait-time
Standardized patient actors	▪ Standardized clients make unannounced visit to subset of clinics and complete a checklist	▪ Assess implementation fidelity by documenting type of services offered, quality of services received, and provider attitudes
Random blood draw	▪ Dried blood spots collected at ~10% visits on persons using PrEP	▪ Objective assessment of PrEP adherence (tenofovir levels)
Nested observational cohort	▪ Women identified as eligible for PrEP, both who initiate and those who decline	▪ Detailed individual-level outcomes: HIV risk behaviors, HIV risk perception, HIV incidence, HIV prevention decision making, contraception use, stigma, STI burden, PrEP retention, and reasons for discontinuation

#### Abstraction of program data

Clinical records in FP clincs are documented on standardized MOH FP register which collects program data on age, contraception method use, women HIV status, and cervical counseling. In collaboration with the Kisumu county health team, new indicators on screening for HIV risk and PrEP eligibility as part of our goal to streamiline simple but scalable HIV porevention program indicators. For persons who initiate PrEP, Kenya MOH clinical encounter form (PrEP Card) is used for clinical monitoring and program evaluation. Dedicated project staff will weekly abstract program data (e.g., number women screened for HIV risk and PrEP initiations) and individual clinical records from these standard MOH tools. Individual data will be used to characterize PrEP screening, PrEP eligibility and initiation, HIV risk profiles of PrEP initiators, self-reported adherence, retention, HIV infection, and side effects.

#### Application of the RE-AIM framework

We will conduct rigorous mixed-methods evaluation using the RE-AIM framework to gain a comprehensive understanding of how effectively PrEP implementation is integrated in FP systems and program impact (Table 2). The RE-AIM framework guides evaluation of the public health impact of complex interventions and can provide actionable information on which to base ongoing and post-trial expansion planning. For this project, Reach will be defined as the proportion and subgroups of women accessing FP services who are screened for and uptake PrEP; Effectiveness will be assessed at individual level as 1) PrEP adherence, quantified by tenofovir levels in dried blood spots, and 2) women staying HIV free; Adoption will be assessed at the clinic and provider level defined as the proportion of 1) clinics approached who implement the intervention 2) targeted providers who are trained and implement PrEP thereafter; Fidelity to PrEP delivery will be assessed as proportion of core components delivered per Kenya PrEP guidelines; and Maintenance, will be the number of clinics delivering PrEP at 6 and 12 months after the intensive technical assistance

**Table 2:** Application of the RE-AIM framework to evaluate integration of PrEP delivery in into public health FP clinics

Domain	Measurement level	Project specific outcome measures
Reach	Individual	▪ Proportion of women screened for HIV risk and PrEP ▪ Number and proportion of at-risk persons initiated on PrEP
	Clinic	▪ Characteristics of implementing clinics
Effectiveness	Individual	▪ Incident HIV infection among PrEP users ▪ Detectable tenofovir levels in randomly collected DBS from PrEP users ▪ Client opportunity costs (e.g., clinic wait time)
	Program	▪ Cost and budget impact from the pragmatic perspective
Adoption	Individual	▪ PrEP continuation
	Clinic	▪ Number and % of clinics initially approached implementing PrEP in FP clinics ▪ Number and % of trained clinical FP staff who delivered PrEP at least once
Implementation	Provider	▪ Number and % of trained appropriately initiated on PrEP ▪ Consistency of implementation across staff (assessed by patient actors) ▪ Opportunity costs (e.g., Workload, impact on other services)
	Clinic	▪ Number of clinics regularly completing MOH PrEP M & E tools and report PrEP ▪ Number and proportion of clinics in which screening for HIV risk and PrEP regularly documented. ▪ Proportion of women testing is documented
Maintenance	Individual	▪ 3- and 6-month PrEP continuation rates
	Clinic	▪ Number of FP clinics implementing PrEP FP 6 months, 12 months after scale back of technical assistance ▪ Number of clinics with PrEP listed in the service charter

#### Clinic readiness to implement PrEP in FP clinics

At each clinic prior to implementation, we will administer a validated organizational readiness for change ORIC psychometric tool [22] to clinic managers and providers to assess for determinants of readiness to implement, including provider knowledge and confidence to provide PrEP, perception of resource availability, and situational factors including timing of the program. We will define facets of readiness (i.e., change commitment and change efficacy), their immediate determinants, and how they impact implementation of PrEP. We will use this data to understand clinic-level variations in implementation performance at both high and low performing clinics, and how they affect implementation outcomes.

#### Clinic and client flow mapping

We will conduct workflow mapping at all sites to identify potential bottlenecks, differences and commonalities among sites at baseline and annually. We will solicit provider engagement in defining new flow maps to accommodate new tasks and patient flows. Current and future flow maps will be created for each site to demonstrate new pathways.

#### Qualitative interviews to understand delivery

We will prospectively conduct qualitative interviews to rigorously understand key influences on integration of HIV prevention and PrEP in FP clinics, at the level of individual women (clients), healthcare providers, the organization (i.e., clinic), and the health system (policy key-informants). Interviews will use the Consolidated Framework for Implementation Science Research (CFIR) [23] to guide development of data collection instruments, data analysis, and reporting of findings.

Qualitative interviews with women will assess women’s experiences accessing and using (or not using) HIV prevention services, including PrEP in FP clinics, confidence in the health system, decision-making processes, partnership dynamics, and stigma and community norms around HIV and FP. Because our overarching goal is development of a sustainable HIV prevention program broadly, not specific to PrEP, stratified purposive sampling will be used to ensure that the overall sample represents relevant sub-populations of women including [24, 25]: 1) women who have risk factors for HIV but choose not to initiate PrEP, 2) women who choose to initiate PrEP, and 3) women who initiate PrEP but later discontinue use.

Qualitative interviews with healthcare providers at FP clinics (clinic managers, counselors, nurses, doctors) and policy key informants (national level and County level) will be conducted during baseline, early implementation and maintenance phases to understand contextual enablers and barriers of implementation acceptability, feasibility, and fidelity. We will specifically evaluate knowledge and confidence delivering PrEP, perception of the impact of PrEP delivery on current FP service provision, and suggestions for efficient integration approaches in the context of FP clinics. Analysis of personal experiences with integrated FP and PrEP delivery will be used to identify determinants of successful implementation and innovative strategies that can be harnessed for future scale-up.

#### Quantitative surveys

We will use quantitative tools including cross-sectional exit surveys among women to complement qualitative interviews. Surveys will focus on understanding HIV risk perceptions, services offered or expected to be offered, quality of care and women satisfaction with services received, and women’s experiences with the health systems. Cross-sectional surveys will be conducted during baseline period and at least annually by project dedicated staff, as clients exit the facility. These surveys will help obtain important data that cannot be obtained from program tools. We will also use a quantitative tool for CFIR inner settings [26] with clinic-managers, in-charges, and frontline healthcare providers during intervention implementation phase to gain deep understanding of clinic-level factors that impact implementation. Data from provider surveys will provide further insights into characteristics of low and high performing clinics.

#### Standardized patient actors to assess implementation fidelity

We will prospectively document the extent to which the core components of the PrEP implementation are implemented as planned. We will examine fidelity across dimensions of adherence, exposure, quality of delivery, provider responsiveness, and program differentiation. We will assess and document factors that affect fidelity, including characteristics of providers, clinics, women, the FP or community context, and program support systems (e.g., County supervision, technical assistance). Specifically, we will use unannounced standardized patient actors to measure fidelity of the implementation of comprehensive HIV prevention services, including PrEP delivery (i.e., counseling and screening for HIV risk factors, HIV testing, PrEP provision). Patient actors will visit a subset of clinics for two consecutive visits, once to screen for HIV risk and PrEP, and a second time to refill PrEP. At the end of each unannounced visit, the standardized patient will complete a checklist to document offered services, quality of services received, provider attitudes, and visit duration.

#### Random blood draw to evaluate PrEP adherence

Among women who initiate PrEP, adherence will be objectively evaluated by tenofovir levels measured from dried blood spots collected at a subset of randomly selected visits (~10-20%). Adherence will also be measured using pharmacy refill records (i.e., picking up each new PrEP supply), and self-report (e.g., self-rating, captured on the PrEP delivery tool).

#### Nested observational cohort for individual-level outcomes

Within the programmatic setup, we will establish an open cohort of HIV-uninfected women to measure individual-level outcomes on incident HIV and STIs, HIV prevention behavior, PrEP use, and adherence. The cohort will enroll up to 900 women, including women who do and do not choose to initiate PrEP. Eligible women must be HIV negative, sexually active, age 15 years and older, and have at least one behavioral factor defined by the Kenya PrEP guidelines to indicate a substantial ongoing risk of acquiring HIV (as described in the Subsection on Screening for HIV risk and PrEP). Informed consent will be obtained for all research procedures not related to routine care. Cohort participants will complete visits at one month and then quarterly after enrollment for up to 24 months with HIV testing and a brief questionnaire on sexual behaviors, FP and prevention methods use, HIV and STI risk, partner relationship, stigma, mental health, and for women initiated on PrEP, dried blood spots will be collected for objective assessment of adherence. All cohort activities will be conducted by project-dedicated staff. In addition to promotion of standard screening and treatment for STIs across all clinics, we will perform baseline etiological testing for curable STIs (Chlamydia trachomatis and Neisseria gonorrhea) to document the burden for women enrolled in the cohort. Urine from cohort follow-up visits will be archived for future STI testing.

#### Health economics studies

We will conduct activity-based costing, staff interviews, and time and motion studies to estimate the cost and model the budget impact of integrating PrEP provision in FP clinics on annual basis, over a 5-year horizon. Using data on the incremental cost estimated during the implementation phase, we will assess the budget impact on the Kenya MOH and Kisumu County budgets for integrating the interventions incorporating the eligible population size, and current MOH and Kisumu budget expenditures, and evaluate for scenarios with current and future HIV prevention methods mix. We will consider direct program costs to ensure that measurements of MOH costs reflect the opportunity cost of the resources used in delivering services. Program costs will also be collected from the project budget, public health clinic budgets, government reports, and the health economics literature, as we have done in previous work [27–30]. The primary analysis will be from the programmatic perspective, with a secondary analysis from the societal perspective to account for client opportunity, affordability, and financial costs. Health outcomes will include incident HIV cases and disability-adjusted life years averted.

#### Data management and confidentiality

All abstracted health and patient data are kept secure and confidential through adherence to institutional policies and procedures for securely storing, maintaining, and updating health record information. All health record and patient data are securely stored on password protected and encrypted servers and these data will not be released externally except under specific data sharing agreements. All study results will be presented in aggregate, and no individual patient or provider will be identifiable.

### Analysis

#### Quantitative data:

The primary analysis will use individual-level data from the SWCRT (counseling on HIV risk and PrEP, PrEP initiation, provision of contraception, and type of contraception provided), individual-level data from the blood sample cohort (PrEP adherence), individual-level data from the longitudinal research cohort, and clinic-level data from the SWCRT (HIV diagnosis). Descriptive statistics of the outcomes will be summarized by study step (baseline, step one, step two or step three) and clinic within each step, as well as overall.

#### Outcomes and Inference

The co-primary outcome to evaluate the intervention effect will be: 1) proportion of women screened for on HIV risk and PrEP; and 2) PrEP uptake. Analyses of the remaining outcomes (knowledge of partner HIV status, PrEP adherence, HIV diagnosis, provision of contraception, type of contraception provided, and STI screening and treatment) will be descriptive and will only involve individuals enrolled (for individual-level outcomes) and data collected (for clinic-level outcomes) after the intervention was initiated. The relative risk (RR) for the effect of the intervention on each outcome will be estimated from the individual-level data (based on baseline and steps one to three) using a generalized estimating equations (GEE) approach [31, 32]. Specifically, we will fit a modified Poisson GEE, i.e., a generalized linear model with log link and robust standard errors [33] to account for clinic clustering in outcomes and to appropriately model a binary outcome, allowing estimation of the relative risk. The predictor of interest will be the intervention status of the site at the time the participant was enrolled (control or intervention). The model will adjust for the partial confounding by time inherent in this design by including the step during which the participant enrolled as a categorical variable in the model. Using the implementation and maintenance phases, we will conduct secondary analyses using similar methods to estimate changes in both clinical and implementation outcomes during the maintenance phase compared to the intensive phase to quantitatively ascertain level of institutionalization. Importantly, we will use data from the maintenance phase to descriptively explain implementation outcomes. In sensitivity analyses, we will also repeat the above analysis but allow for the intervention effect to vary with time; in other words, we will include an interaction between treatment assignment and step, where step is modeled as a categorical variable [34]. This will allow us to evaluate possible variations in treatment effect over time given that the intervention may change over time due to feedback throughout the study period.

#### Qualitative data

Qualitative data (i.e., technical assistance reports, individual interviews, and observation reports) will be used to interpret and explain contextual influences on individual RE-AIM dimensions or patterns of results across implementation phases and/or clinics. We will use rapid qualitative analysis approaches to obtain real-time information [35–37] that can directly inform study implementation*.* In addition, we will use a combination of conventional and directed content analysis approaches [38] to identify determinants of high and low performing facilities during early implementation and maintenance phases. Coding and analysis will be performed in Atlas.ti. Qualitative data sources (transcripts and reports) will be iteratively coded using a structured codebook developed deductively from the constructs within CFIR and inductively from open-coding. Coding will be conducted by multiple team members, and each data source reviewed by at least two members of the research team to ensure consistency of text segmentation and code application. Queries will be used to abstract related concepts and generate preliminary themes. Preliminary themes will be grouped together into larger themes through group deliberative processes. We will triangulate qualitative and quantitative data together to answer relevant questions about delivery process and outcomes simultaneously. Specially, we will characterize common themes and profiles of low and high performing facilities as determined by the proportion of women screened, initiated, and retention on PrEP.

### Protocol amendments

This protocol (version 1.0, October 27, 2020) was registered with ClinicalTrials.gov on December 14, 2020 (NCT04666792). Amendments to the study protocol will require approval from the Ethics Review Committee of the Kenyatta National Referral Hospital- University of Nairobi and the Human Subjects Division of the University of Washington. Any amendments will be communicated via trial registration updates and reported in any published manuscripts associated with the study as necessary.

### Dissemination plan

The study team for this award is committed to public dissemination of results of the project to local stakeholders in Kenya, the global scientific community, and global policymakers. Dissemination of study results will follow principles of good participatory practice. Results will be published in conference abstracts and peer-reviewed journals. Study results will be disseminated through presentations to global and local stakeholders and policymakers in Kenya, including working in close collaboration with the Kenyan Ministry of Health to help foster immediate translational impact. Authorship eligibility will be determined according to the International Committee of Medical Journal Editors recommendations.

### Project status

The project activated in March 2021 and implementation is currently ongoing and expected to end in 2024.

## Discussion

As PrEP implementation gradually expands in Africa, initial delivery points have centered in HIV care centers focused on serodiscordant couples, “drop-in” centers, and youth-friendly “safe spaces,” for key populations. For women, access has been limited mostly through a few demonstration projects in FP and antenatal clinics.

The FP Plus project offers a timely opportunity to move from small-scale PrEP demonstration projects to full-scale service delivery for women in Africa. Since the 2015 WHO global recommendation for PrEP was released, understanding how to rapidly and efficiently translate this effective prevention intervention into real-world settings has been the priority, but most work in Africa remains focused on controlled demonstration projects, mostly in research clinics. We have pushed beyond this boundary with the PrIYA project, a demonstration pilot project that has shown that it may be feasible to integrate PrEP in FP systems. In this study we move to the next phase and evaluate whether fully integrating PrEP provision in FP clinics as a one-stop location for women-centered health care services can be done successfully at scale with implementation science evaluations. Our study leverages existing infrastructure and expertise of FP providers in counseling for sexual history and program delivery on a variety of contraception methods, to embed clinical assessment and PrEP provision in combination HIV prevention package. Using current health system, staffing and supply chain and has tremendous potential to address barriers that women face for PrEP access, including lack of time, cost, and stigma of visiting a facility solely for HIV prevention. With more efficient PrEP integration, there is opportunity to improve FP care and optimize women-centered care with a one-stop approach to clinic. The implementation methods and lessons learned through our novel FP-based PrEP care pathway will provide information on sustainable integrated service delivery that can be translated to other African settings. Importantly, the application of rigorous implementation science approaches to understand implementation will be valuable for advancing other emerging novel HIV prevention modalities into real-world delivery.

In Kenya’s devolved government structure, the MOH sets and guides national policy, but implementation of policy and public health services is under the county governments. Our proposed work aligns with the Kenya MOH and Kisumu County priority goals, which makes the probability of impact high. In 2016, Kenya MOH developed a national PrEP implementation framework [21], and national PrEP roll-out was officially launched in May 2017, making it the first African national PrEP program. Because of the Kenyan governments’ strong support for PrEP as an HIV prevention intervention, Kenya is an incubator for research on innovative PrEP delivery models. Successful implementation of PrEP interventions in Kenya has the potential to influence the delivery of PrEP in other African settings. This work will inform implementation of similar interventions elsewhere and facilitate interpretation of intervention outcomes.

## Data Availability

The datasets generated from this protocol will be available after the primary analysis by contacting the International Clinical Research Center at the University of Washington (icrc@uw.edu).

## Abbreviations

ART: Antiretroviral therapy
CFIR: Consolidated framework for implementation science research
DMC: Data monitoring committee
FP: Family planning
HIV: Human immunodeficiency virus
KEMSA: Kenya medical supply authority
MOH: Ministry of Health
NASCOP: National AIDS & STI control programme
PrEP: Pre-exposure prophylaxis
PrIYA: PrEP implementation in young women and adolescent girls
RE-AIM: Reach, effectiveness, adoption, implementation, maintenance
SRH: Sexual and reproductive health
STI: Sexually transmitted infection
SWCRT: Stepped-wedge cluster-randomized trial
TA: Technical advisors
TWG: Technical working group
WHO: World health organization
